# Supplementary material for: Cryo-EM structure of human PAPP-A2 and mechanism of substrate recognition
Source: Commun Chem. 2023 Oct 28;6:234. doi: 10.1038/s42004-023-01032-y (PMC10613257; doi:10.1038/s42004-023-01032-y)
Supplement: Supplementary file 2 — Supplementary information [file 42004_2023_1032_MOESM2_ESM.pdf]

Supplementary information

Cryo-EM structure of human PAPP-A2 and mechanism of substrate recognition

Janani Sridar[1,3], Amirhossein Mafi[1,3], Russell A. Judge[2], Jun Xu[1], Kailyn A. Kong[1], John C.K. Wang[1], Vincent S. Stoll[2], Georgios Koukos[1], Reyna J. Simon[1], Dan Eaton[1], Matthew Bratkowski[1,4†], and Qi Hao[1,4†]

1. Calico Life Sciences LLC, South San Francisco, CA, 94080, USA
2. AbbVie, 1 North Waukegan Rd, North Chicago, IL 60064 USA
3. These authors contributed equally
4. These authors jointly supervised this work

[†]Correspondence: mbratkowski@calicolabs.com and qhao@calicolabs.com

Supplementary Figures 1-17 and legends
Supplementary Table 1

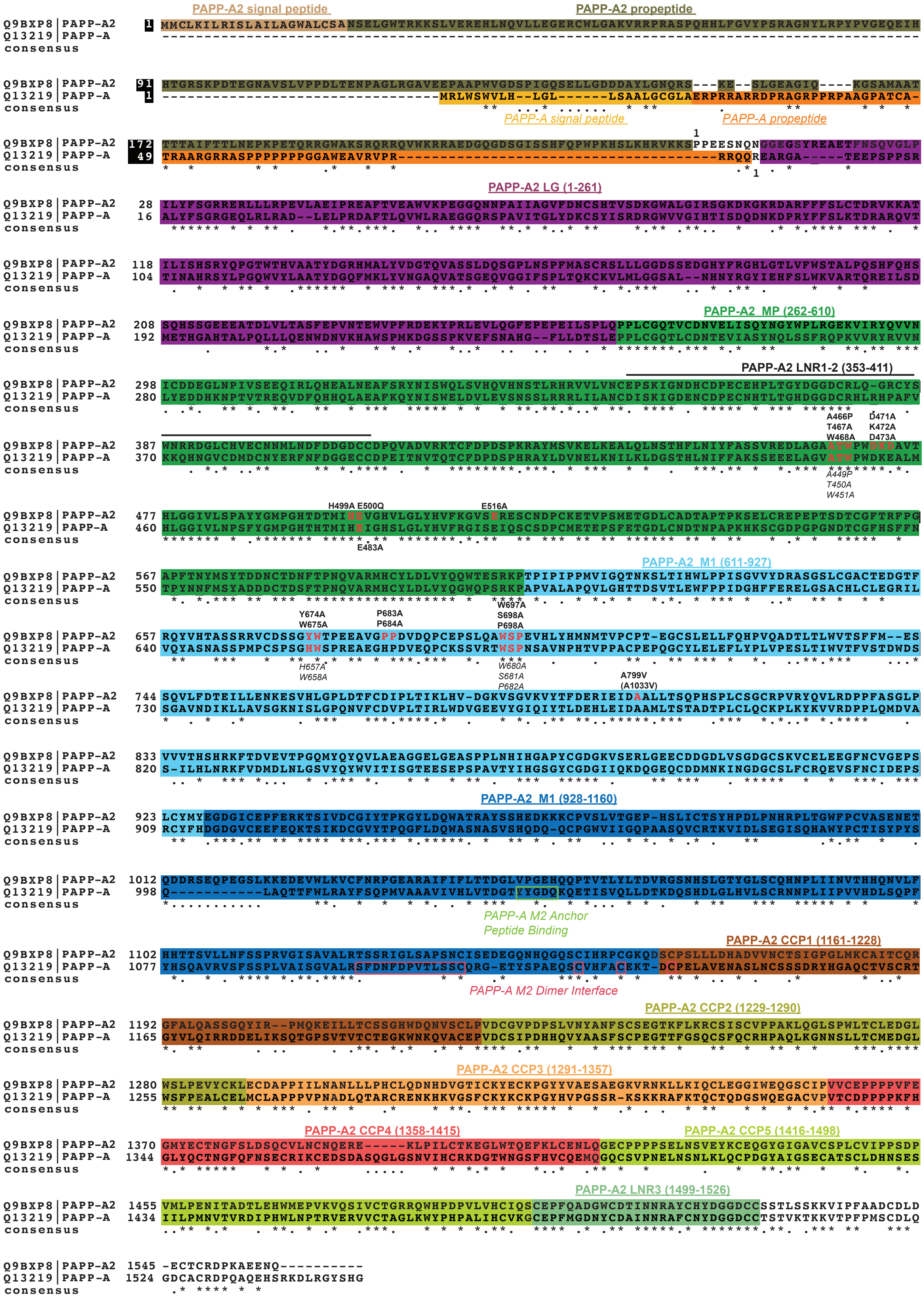

PAPP-A2 signal peptide
PAPP-A2 propeptide
Q9BXP8 | PAPP-A2
Q13219 | PAPP-A
consensus
PAPP-A signal peptide
PAPP-A propeptide
PAPP-A2 LG (1-261)
PAPP-A2 MP (262-610)
PAPP-A2 LNR1-2 (353-411)
A466P
T467A
W468A
D471A
K472A
D473A
A449P
T450A
W451A
H499A E500Q
E516A
E483A
PAPP-A2 M1 (611-927)
Y674A
W675A
P683A
P684A
W697A
S698A
P698A
H657A
W658A
W680A
S681A
P682A
A799V
(A1033V)
PAPP-A2 M1 (928-1160)
PAPP-A M2 Anchor
Peptide Binding
PAPP-A2 CCP1 (1161-1228)
PAPP-A M2 Dimer Interface
PAPP-A2 CCP2 (1229-1290)
PAPP-A2 CCP3 (1291-1357)
PAPP-A2 CCP4 (1358-1415)
PAPP-A2 CCP5 (1416-1498)
PAPP-A2 LNR3 (1499-1526)

Supplementary Figure 1. Sequence alignment of PAPP-A2 and PAPP-A

PAPP-A2 and PAPP-A sequences were aligned with Clustal Omega (version 1.2.4). Asterisks under the sequences indicate residues that are conserved, and dots represent residues that are similar. Domains are color coded. Numbering for the mature protein sequences, which is used in the text, is shown as black script whereas numbering for immature signal peptide and propeptide regions is shown as white script with black highlight. Mutations discussed in the text are shown as red script in the alignment and are labeled in bold above the alignment for PAPP-A2 or italicized below the alignment for PAPP-A. For the PAPP-A2 A799V patient mutation, numbering based on the unprocessed protein is shown in parentheses (A1033V) because this notation in common in the literature. PAPP-A M2 domain residues important for IGFBP5 anchor peptide binding and dimerization are boxed in green and red, respectively.

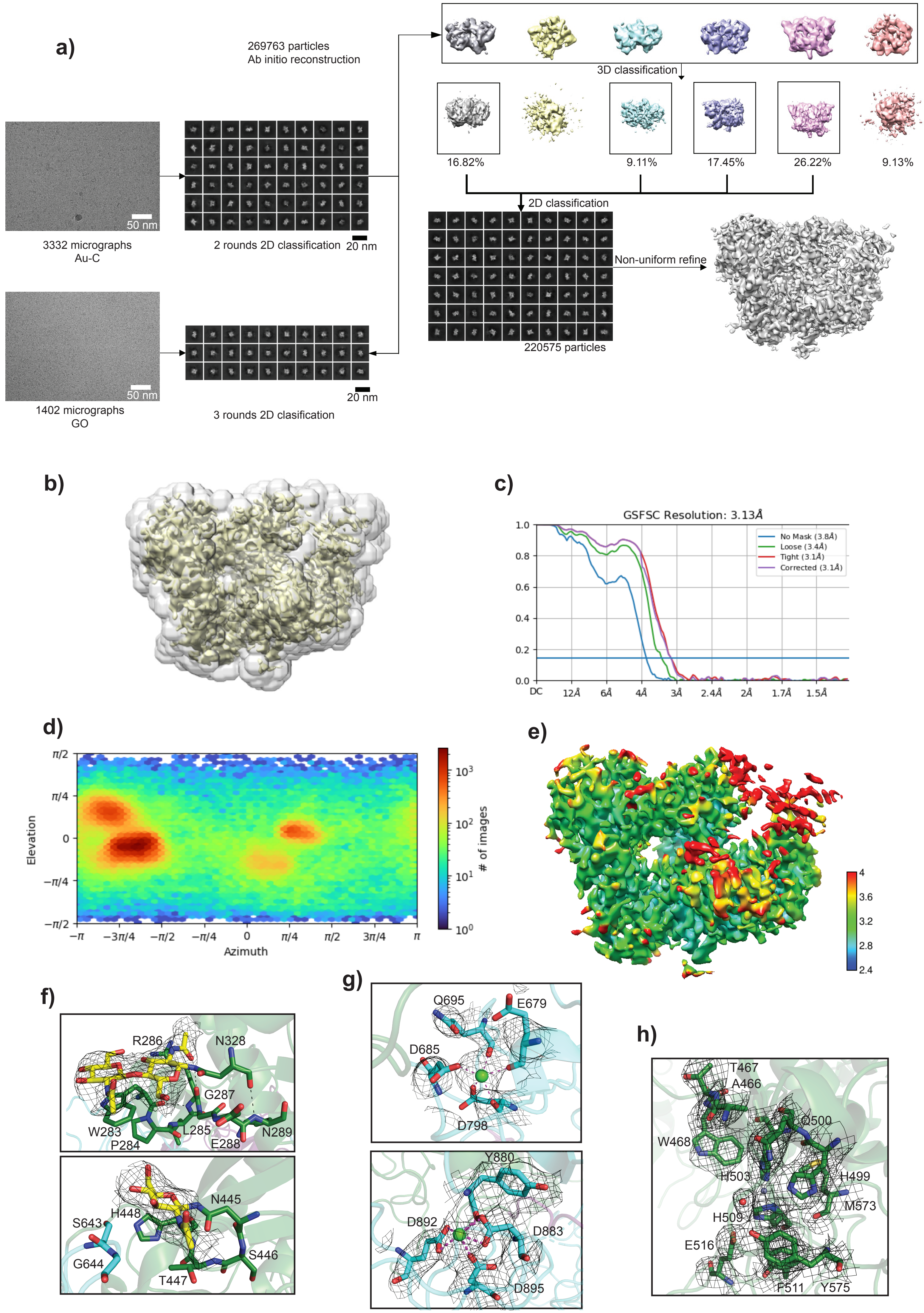
a)
269763 particles
Ab initio reconstruction
3D classification
16.82%
9.11%
17.45%
26.22%
9.13%
2D classification
50 nm
3332 micrographs
Au-C
2 rounds 2D classification
20 nm
Non-uniform refine
220575 particles
50 nm
1402 micrographs
GO
3 rounds 2D clasification
20 nm
b)
c)
GSFSC Resolution: 3.13Å
No Mask (3.8Å)
Loose (3.4Å)
Tight (3.1Å)
Corrected (3.1Å)
1.0
0.8
0.6
0.4
0.2
0.0
DC
12Å
6Å
4Å
3Å
2.4Å
2Å
1.7Å
1.5Å
d)
Elevation
π/2
π/4
0
−π/4
−π/2
−π
−3π/4
−π/2
−π/4
0
π/4
π/2
3π/4
π
Azimuth
# of images
10^3
10^2
10^1
10^0
e)
4
3.6
3.2
2.8
2.4
f)
R286
N328
G287
W283
L285
N289
P284
E288
N445
H448
S643
S446
G644
T447
g)
Q695
E679
D685
D798
Y880
D892
D883
D895
h)
T467
A466
Q500
W468
H503
H499
M573
H509
E516
F511
Y575

Supplementary Figure 2. PAPP-A2 Cryo-EM data processing and structural analysis

a) Cryo-EM processing pipeline showing representative micrographs, initial 2D particle classification, 3D classification followed by a second round of 2D classification based on the best 3D classes, and the final model. **b)** A mask (gray surface) was used for 3D refinement that encompasses the entirety of the PAPP-A2 density (gold). **c)** Gold standard Fourier shell correlation (GSFSC) with cutoff of 0.143 for the final map indicates an overall resolution of 3.13 Å. **d)** Per-particle distribution over azimuth and elevation angles. **e)** Local resolution map with a color gradient from highest (blue) to lowest (red) resolution. **f)** Zoomed-in views of the glycans (yellow sticks) with the MP and M1 domains in green and cyan, respectively. Hydrogen bonds are shown as black dashes. In this figure and Supplementary Fig.2g and Supplementary Fig.2h that follow, map density is shown as black mesh and is contoured to 6.0 sigma. **g)** Calcium binding sites in PAPP-A2 with calcium shown as lime spheres. Metal coordination bonds are shown as magenta dashes. **h)** PAPP-A2 active site with cryo-EM map density shown.

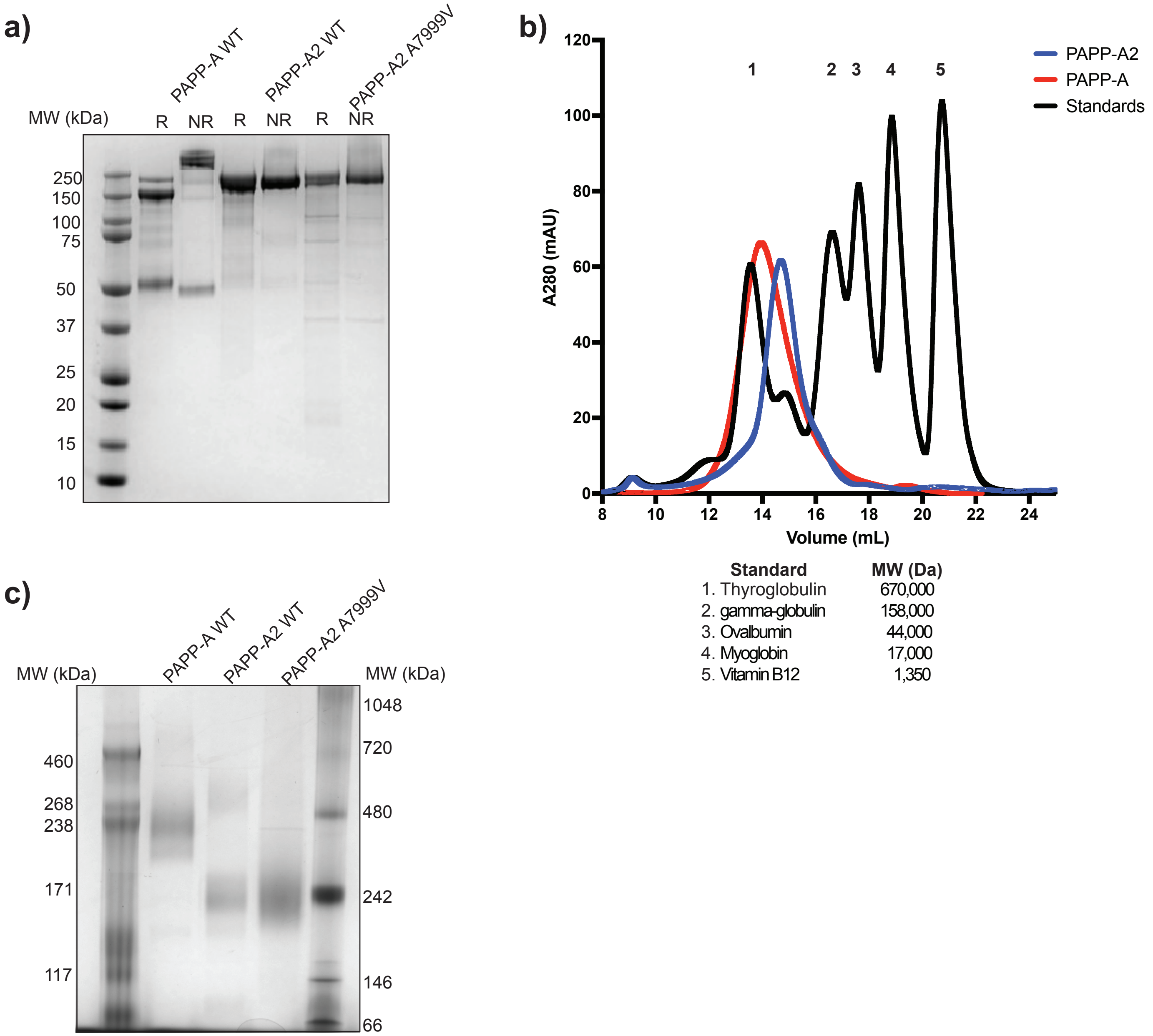

Supplementary Figure 3. Oligomerization analysis of PAPP-A2

a) SDS-PAGE analysis under reducing ("R") and non-reducing ("NR") conditions for wildtype PAPP-A, PAPP-A A799V mutant, and PAPP-A (for comparison) indicate that PAPP-A2 is a monomer. 5 μg of each protein was used for the analysis. **b)** PAPP-A2 is a monomer when run on a Superose 6 10/300 GL column and compared to molecular weight standards (numbers above the chromatograph peaks and the table below) and PAPP-A run in the same manner. **c)** Native PAGE analysis of PAPP-A, PAPP-A A799V mutant, and PAPP-A (for comparison) indicates that PAPP-A2 is a monomer. 2.5 μg of protein was used for the analysis.

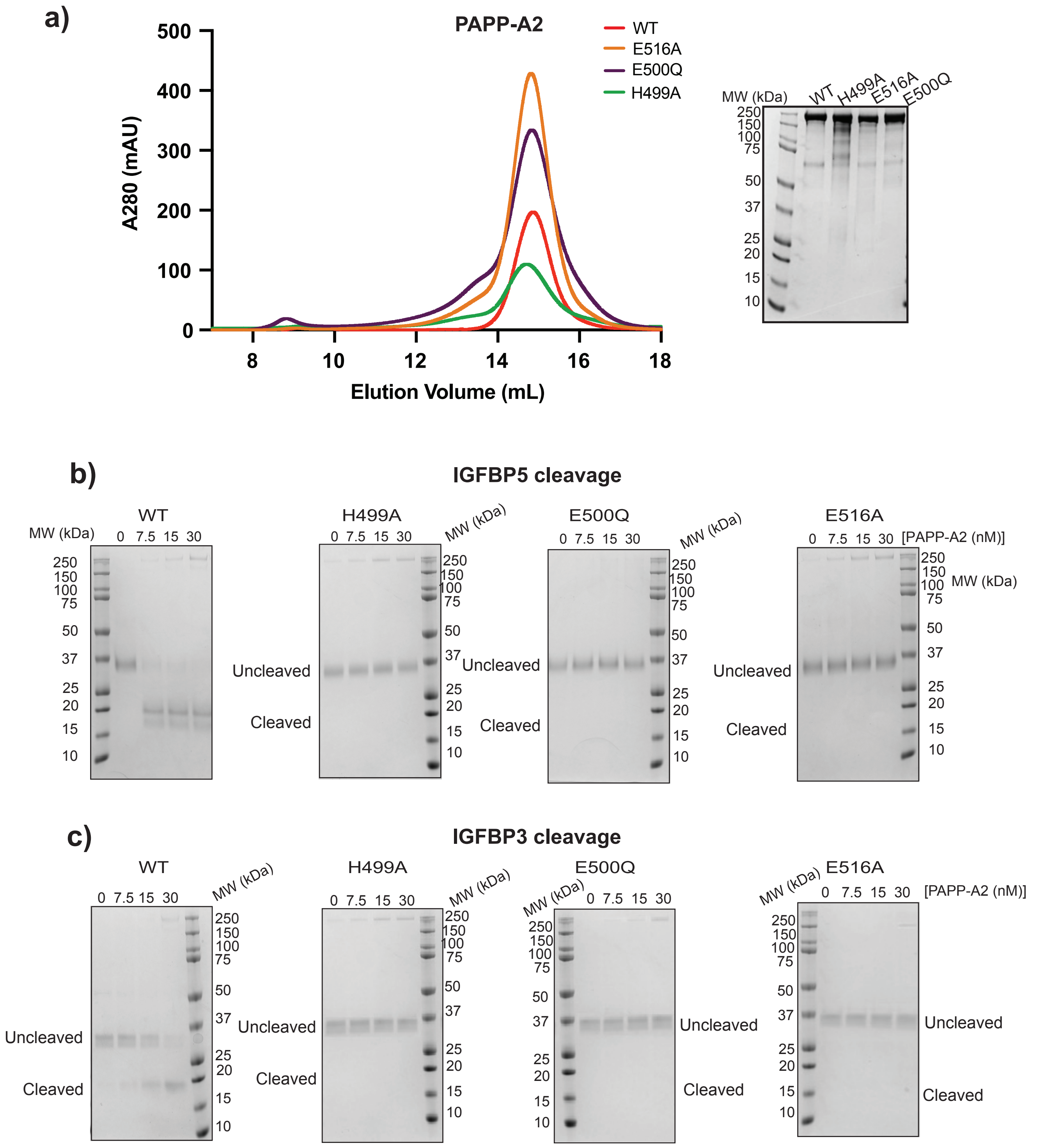

Supplementary Figure 4. Protein quality and a representative assay for PAPP-A2 active site mutants

a) Left, final purification step for wild type and mutant PAPP-A2 proteins by gel filtration on a Superose 6 Increase 10/300 GL column. Monomeric peak fractions were pooled for use in assays. Right, protein analy-sis by denaturing SDS-PAGE followed by Coomassie Blue staining. Please note that the gels shown here and all other gels shown in this report, with the exception of Supplementary Fig.3c and certain lanes in Supplementary Fig.3a, were run under reducing conditions. **b)** Representative, gel-based PAPP-A2 cleav-age assays using IGFBP5 as a substrate. **c)** Representative, gel-based PAPP-A2 cleavage assays using IGFBP3 as a substrate.

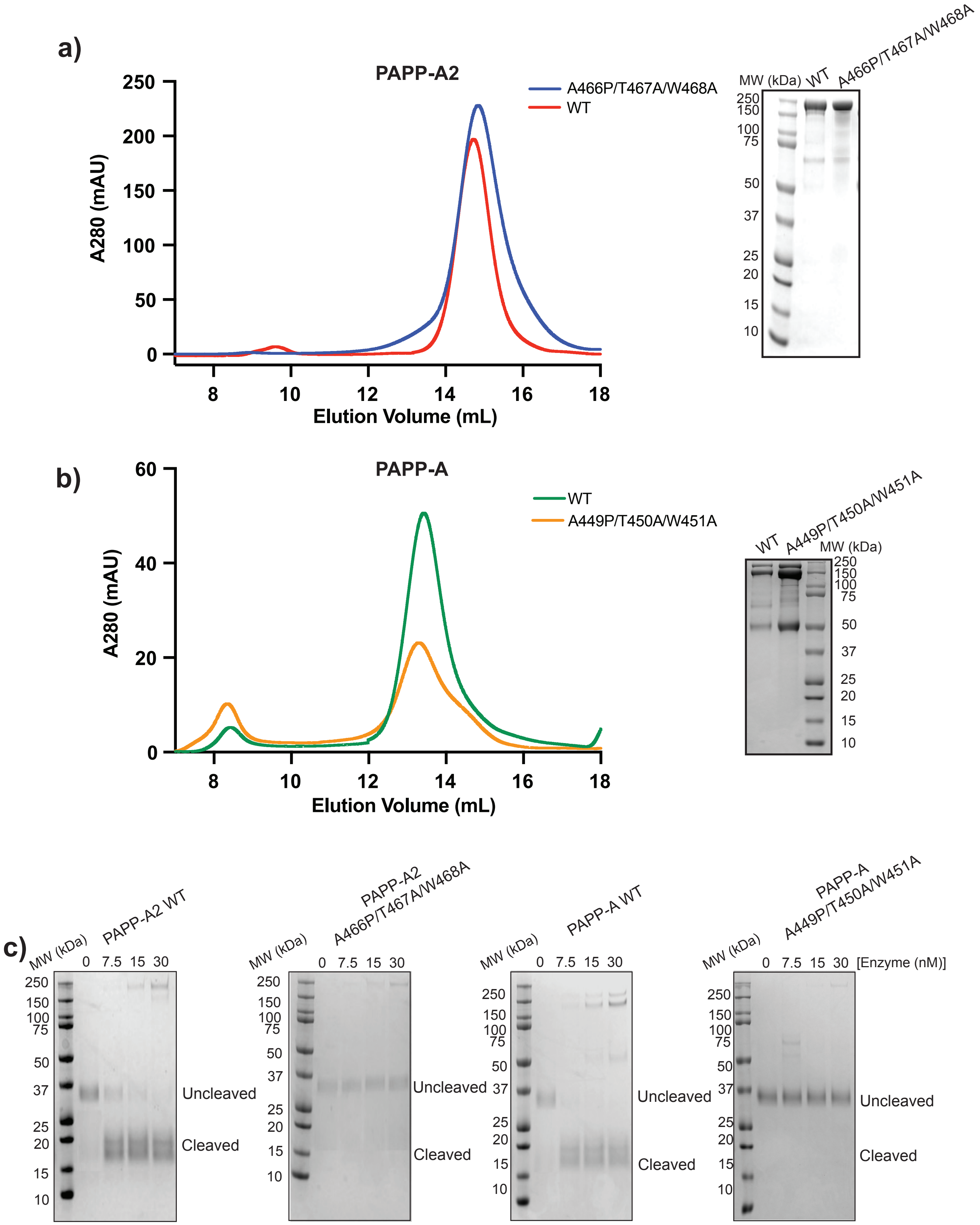

Supplementary Figure 5. Protein quality and representative assay data for PAPP-A2 and PAPP-A peptide-binding mutants

a-b) Left, final purification step for wild type and mutant PAPP-A2 and PAPP-A proteins by gel filtration on a Superose 6 Increase 10/300 GL column. Monomeric peak fractions for PAPP-A2 and dimeric fractions for PAPP-A were pooled for use in assays. Right, protein analysis by SDS-PAGE followed by Coomassie Blue staining. **c)** Representative, gel-based PAPP-A2 and PAPP-A cleavage assays for wildtype IGFBP5.

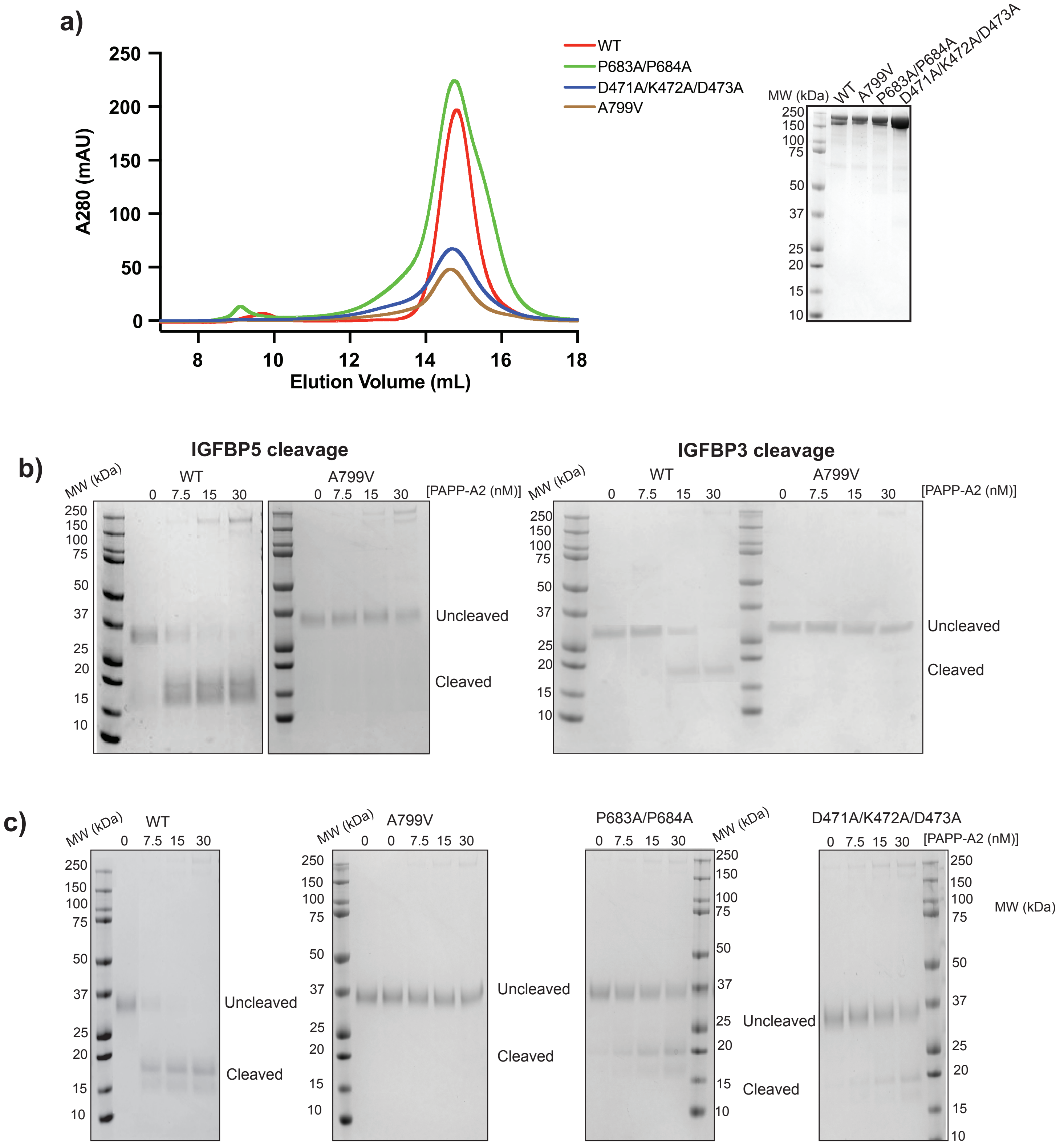

Supplementary Figure 6. Protein quality and representative assay data for PAPP-A2 patient mutation and patch mutants

a) Left, final purification step for wild type and mutant PAPP-A2 proteins by gel filtration on a Superose 6 Increase 10/300 GL column. Monomeric peak fractions were pooled for use in assays. Right, protein analysis by SDS-PAGE followed by Coomassie Blue staining. Note, while some mutants were expressed at lower levels than wild type protein, all proteins were of comparable quality. **b)** Representative, gel-based wild type and patient mutant PAPP-A2 cleavage assays with IGFBP5 (left) or IGFBP3 (right) as a substrate. **c)** Representative, gel-based cleavage assay for PAPP-A2 mutants with a long-range connection to the patient mutation using IGFBP5 as a substrate.

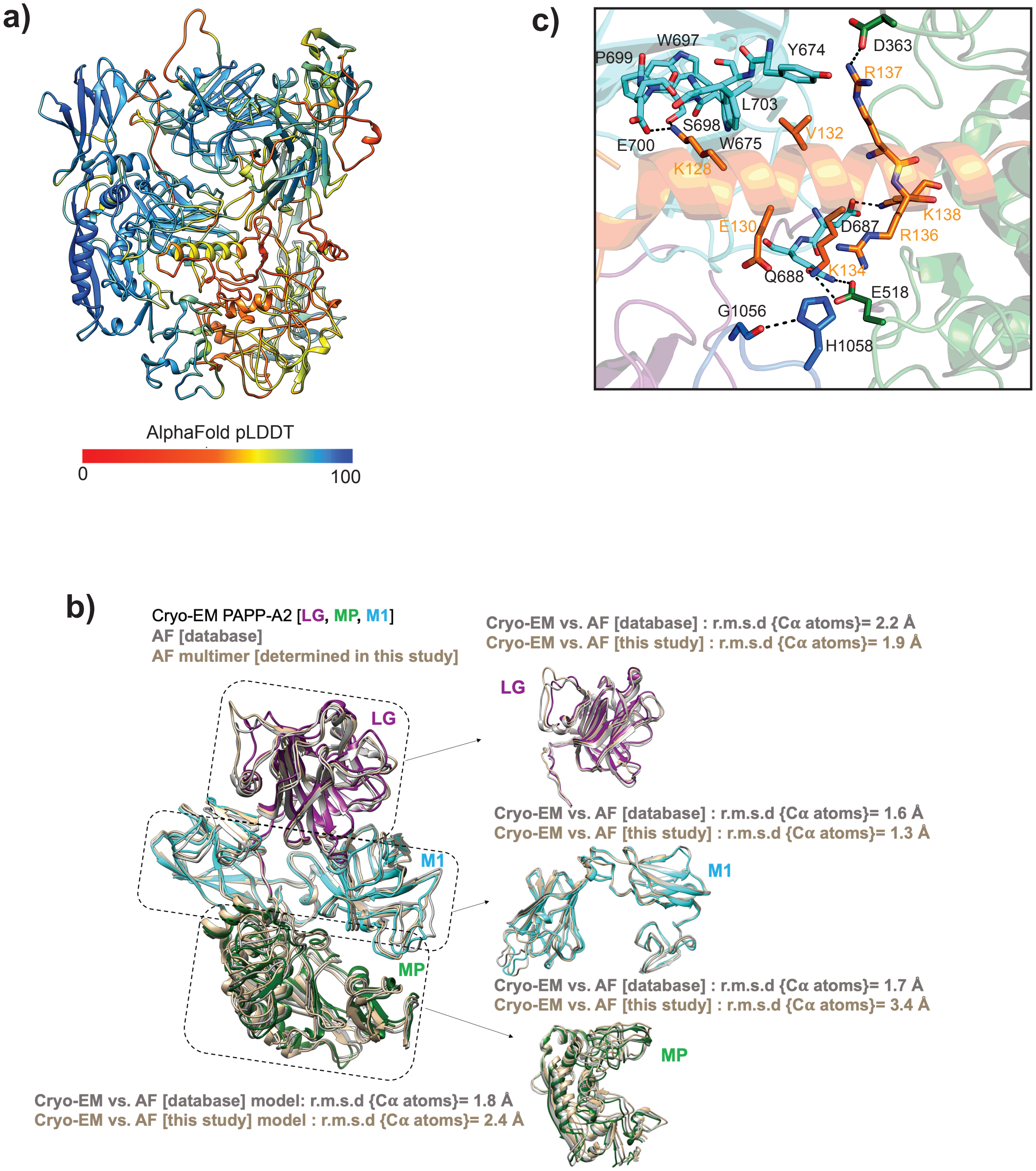

Supplementary Figure 7. PAPP-A2/IGFBP5 ML model and functional validation

a) Machine learning-based PAPP-A2/IGFBP5 complex model (ML-PAPP-A2/IGFBP5) with coloring based on confidence score from red (low) to dark blue (high). **b)** Structural alignment of the PAPP-A2 cryo-EM structure (LG (purple), MP (green), and M1 (cyan), the PAPP-A2 AlphaFold model from the database (gray), and the ML-PAPP-A2/IGFBP5 model determined in this report (tan). **c)** Zoomed-in view of the ML-PAPP-A2/IGFBP5 model interaction site (black box in Fig. 3a) with predicted interactions shown in sticks. PAPP-A2 domains are color-coded as in Fig. 3a and the IGFBP5 anchor peptide is in orange.

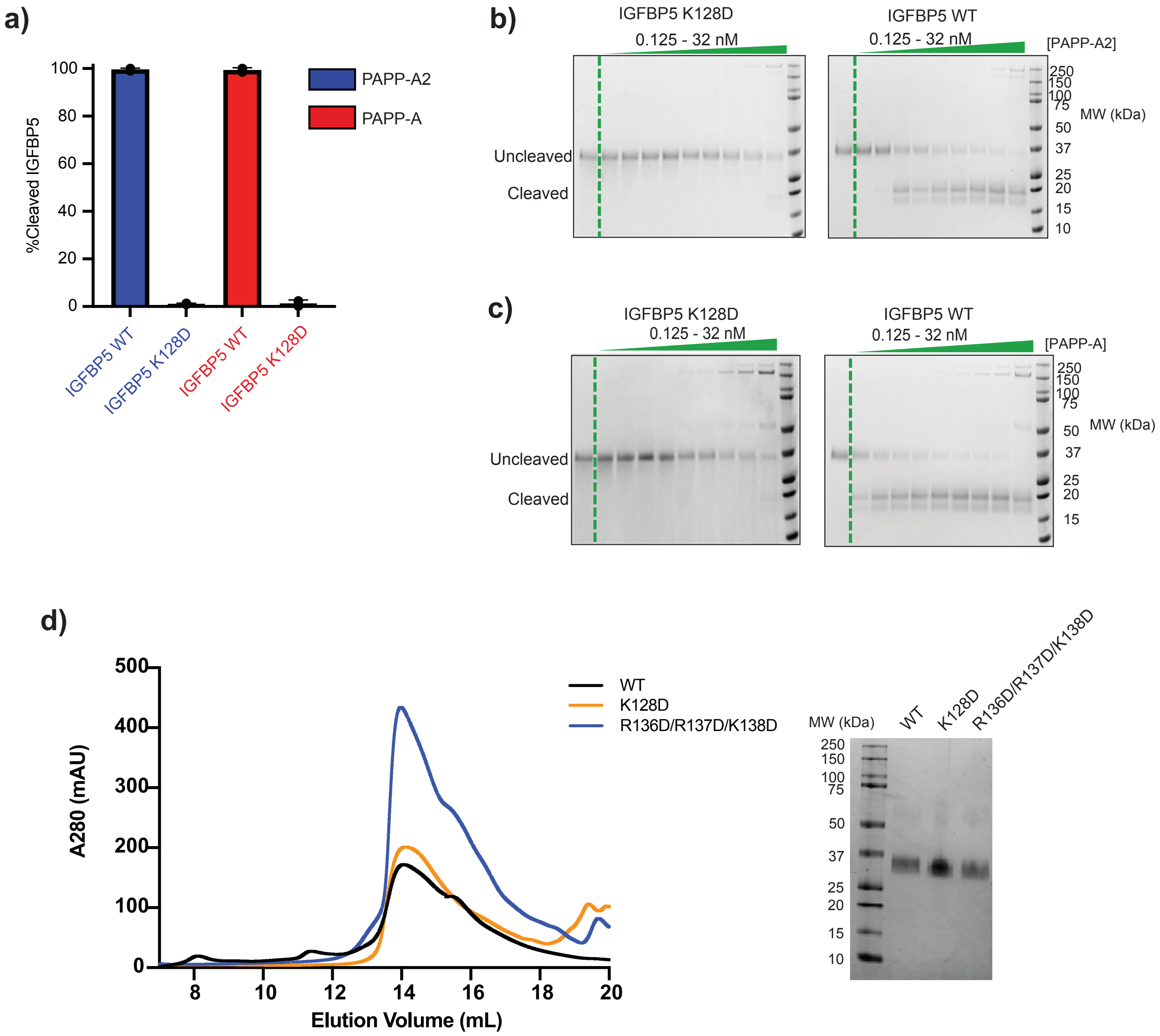

Supplementary Figure 8. IGFBP5 protein quality and representative IGFBP5 K128D cleavage assays
a) Cleavage comparison of PAPP-A2 (blue bars) and PAPP-A (red bars) on wild type and K128D IGFBP5 mutant substrate. The graphed data represents cleavage efficiency at a concentration of 8 nM for both PAPP-A and PAPP-A2. Error bars represent standard deviation of experiments done in triplicates. **b)** Representative cleavage data for wildtype IGFBP5 and K128D mutant for PAPP-A2. **c)** Representative cleavage data wildtype IGFBP5 and K128D mutant for PAPP-A. Two-fold, serial dilutions of PAPP-A2 or PAPP-A were used as indicated by the green ramp. Note that no significant cleavage of IGFBP5 K128D was observed at higher concentrations of PAPP-A2 or PAPP-A. **d)** Left, final purification step for wildtype and mutant IGFPB5 proteins by gel filtration on a Superdex 200 Increase 10/300 GL column. Monomeric peak fractions were pooled for use in assays while trailing shoulders were avoided. Right, IGFBP5 protein analysis by SDS-PAGE followed by Coomassie Blue staining.

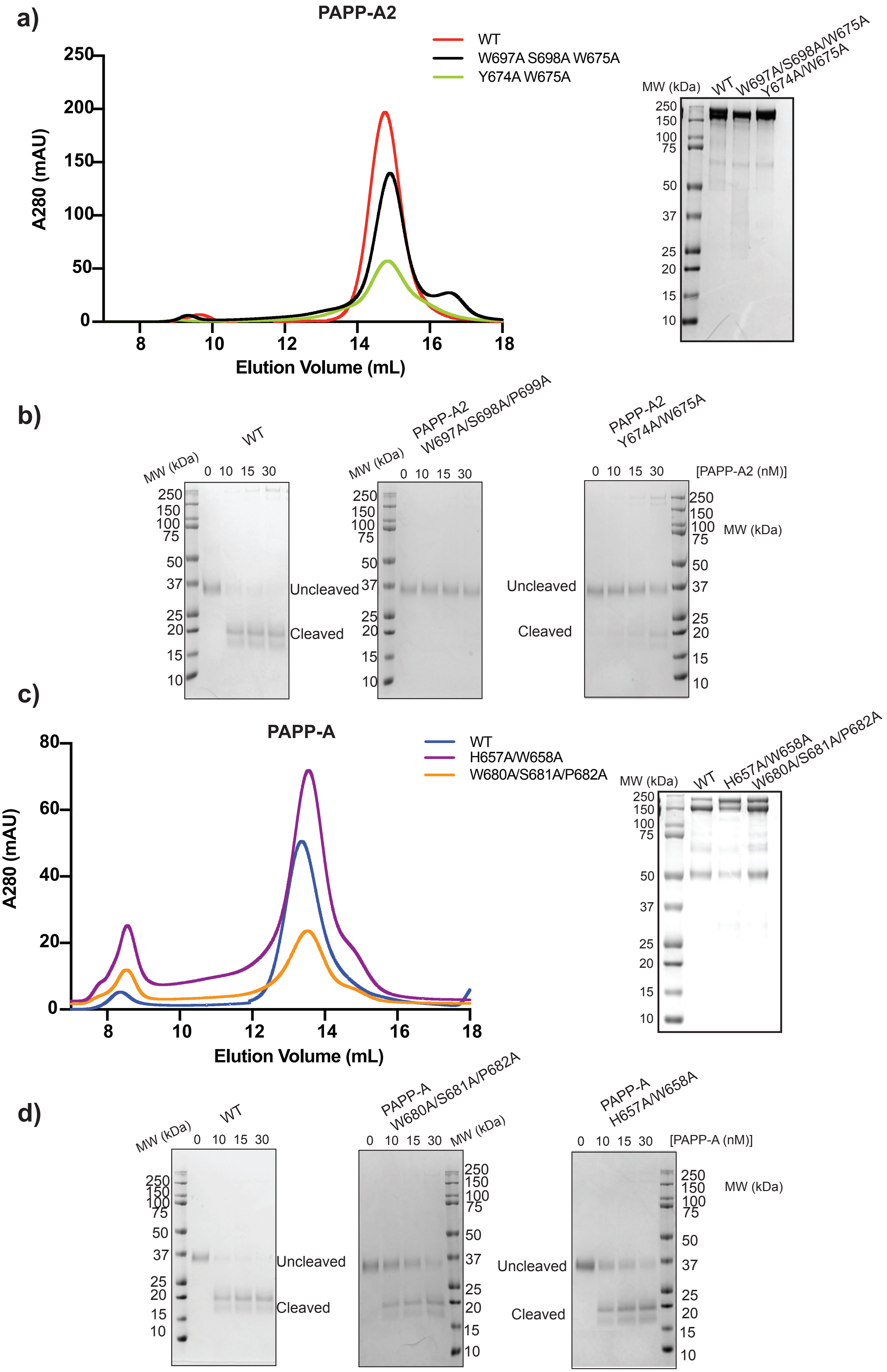

a)
PAPP-A2
WT
W697A S698A W675A
Y674A W675A
A280 (mAU)
250
200
150
100
50
0
8
10
12
14
16
18
Elution Volume (mL)
MW (kDa)
WT
W697A/S698A/W675A
Y674A/W675A
250
150
100
75
50
37
25
20
15
10
b)
MW (kDa)
WT
0 10 15 30
Uncleaved
Cleaved
PAPP-A2
W697A/S698A/P699A
PAPP-A2
Y674A/W675A
[PAPP-A2 (nM)]
c)
PAPP-A
WT
H657A/W658A
W680A/S681A/P682A
A280 (mAU)
80
60
40
20
0
Elution Volume (mL)
d)
PAPP-A
W680A/S681A/P682A
PAPP-A
H657A/W658A
[PAPP-A (nM)]

Supplementary Figure 9. Protein quality and representative assay data for PAPP-A2 and PAPP-A anchor-peptide binding mutants

a) Final purification step for wild type PAPP-A2 and mutants on a Superose 6 Increase 10/300 GL column (left) and protein analysis by SDS-PAGE followed by Coomassie Blue staining (right). **b)** Representative, gel-based wildtype and mutant PAPP-A2 cleavage assay with wildtype IGFBP5. **c)** Final purification step for wild type and PAPP-A and mutants on a Superose 6 Increase 10/300 GL column (left) and protein analysis by SDS-PAGE followed by Coomassie Blue staining (right). **d)** Representative, gel-based PAPP-A cleavage assay with wildtype IGFBP5.

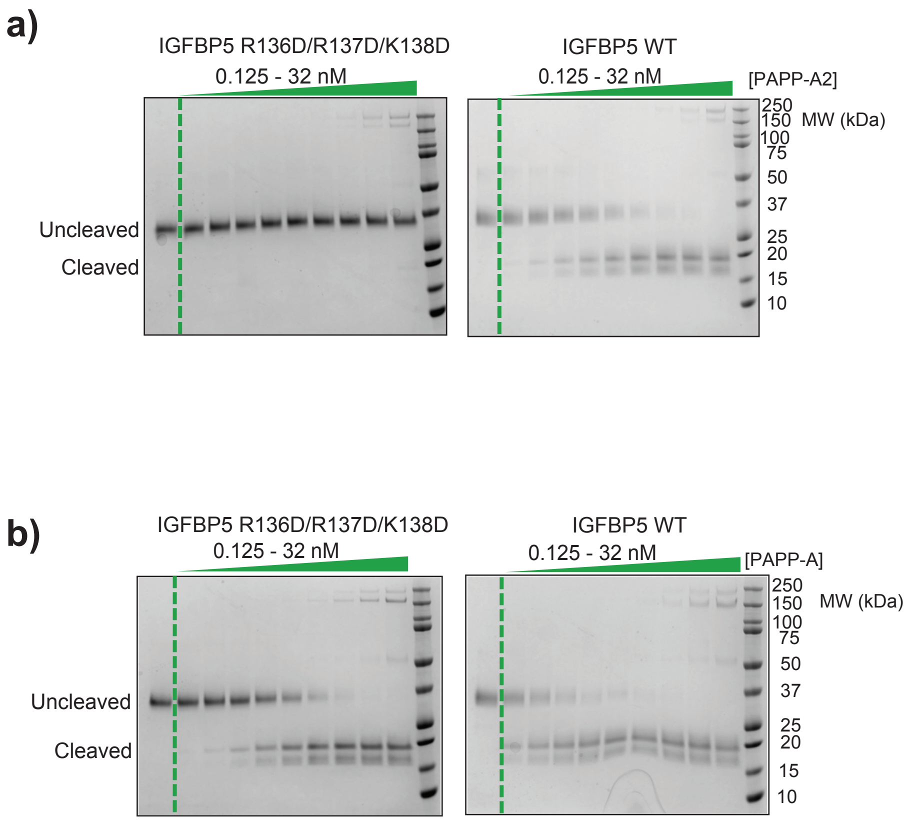

Supplementary Figure 10. Representative IGFBP5 R136D/R137D/K138D cleavage assays

a-b) Representative gel-based assays for PAPP-A2 and PAPP-A with wild type and R136D/R137D/K138D mutant IGFBP5 substrate. Two-fold, serial dilutions of PAPP-A2 or PAPP-A were used as indicated by the green ramp. Protein quality for the IGFBP5 R136D/R137D/K138D mutant is shown in Supplementary Fig.8d.

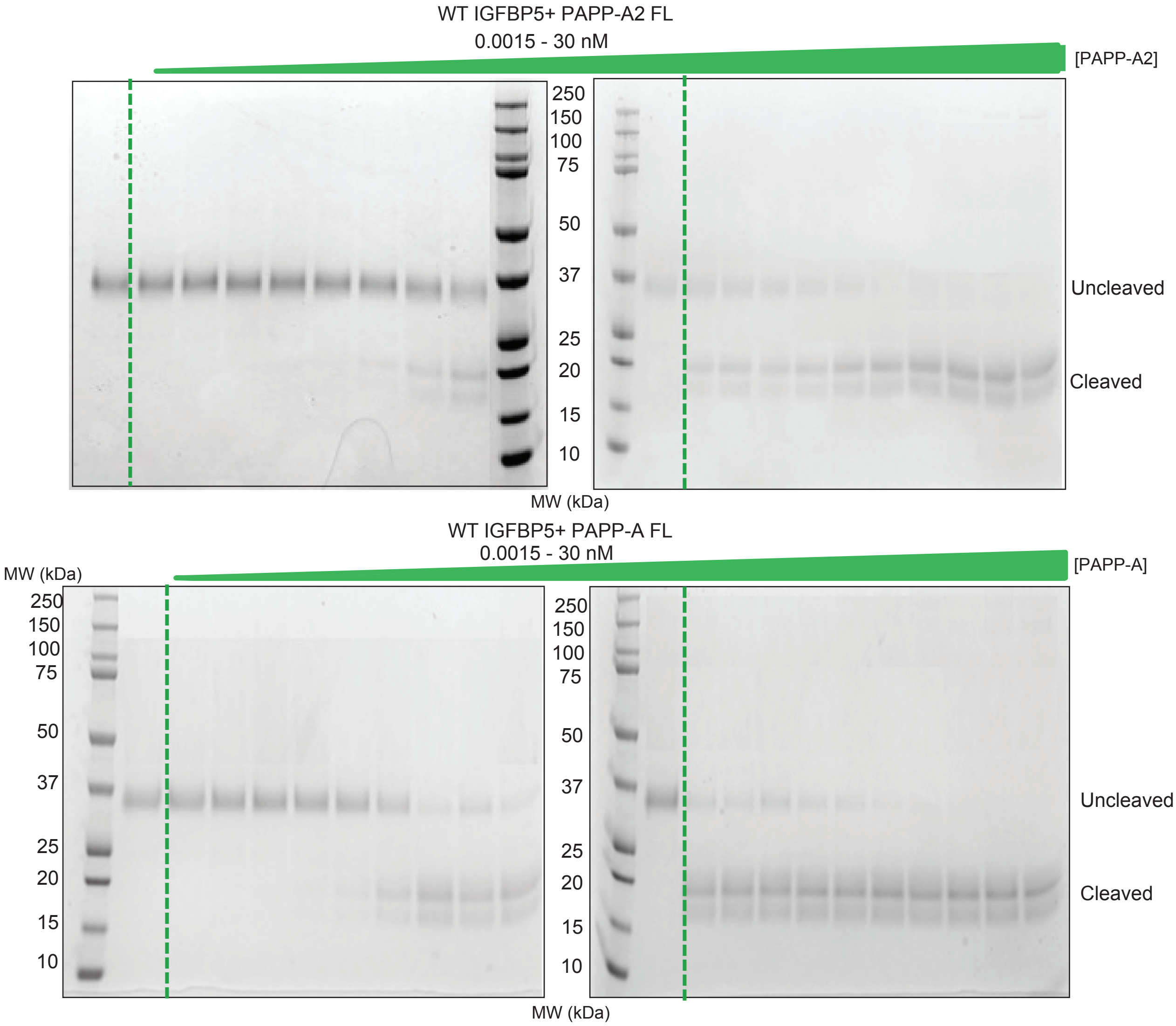

Supplementary Figure 11. Representative PAPP-A2 and PAPP-A comparison cleavage assays

1.5-fold, serial dilutions of PAPP-A2 or PAPP-A were used as indicated by the green ramp.

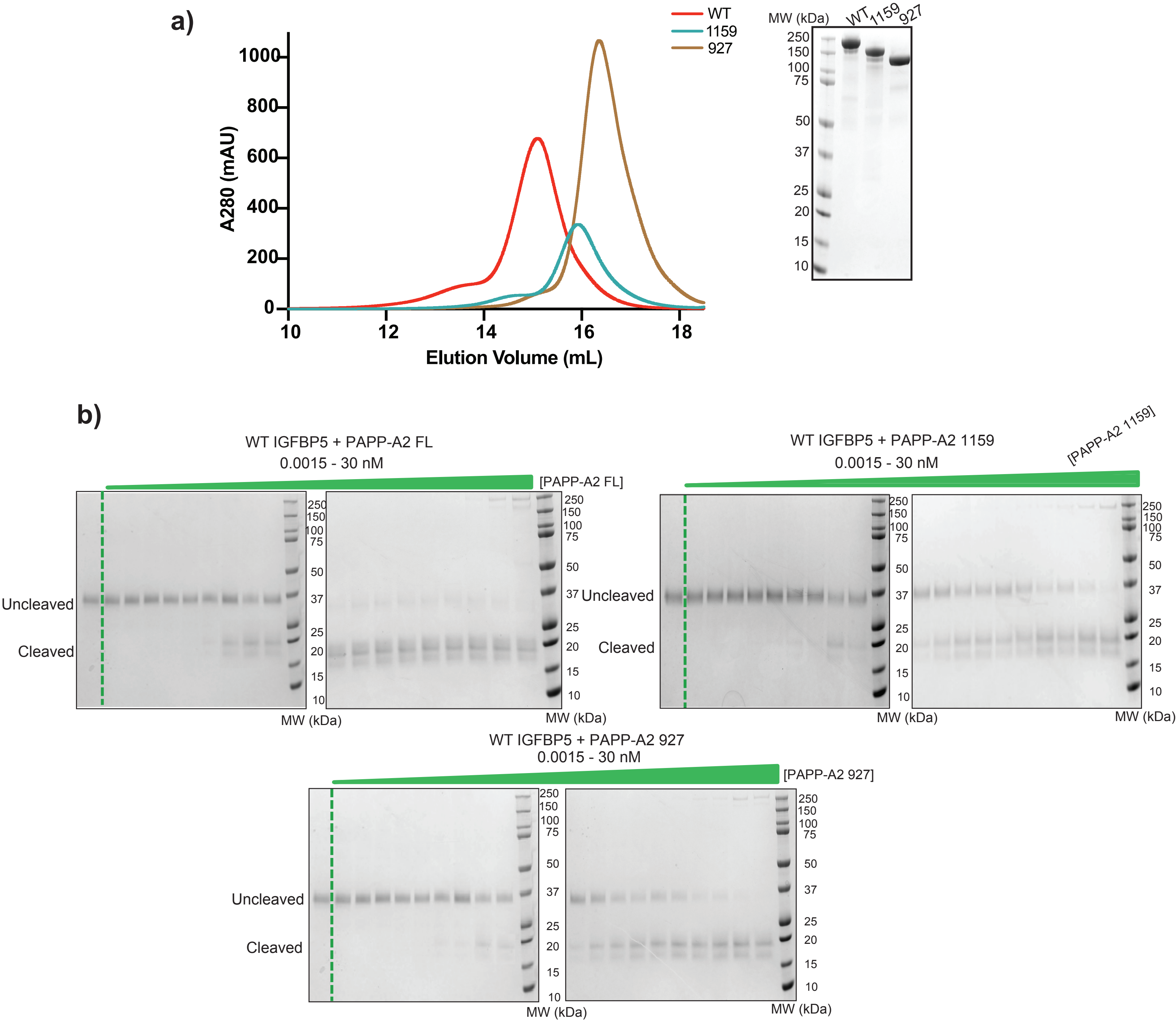

Supplementary Figure 12. Protein quality and representative assay data for PAPP-A2 truncation variants
a) Left, final purification step for wild type and truncated PAPP-A2 proteins by gel filtration on a Superose 6 Increase 10/300 GL column. Monomeric peak fractions were pooled for use in assays. Right, protein analysis by SDS-PAGE followed by Coomassie Blue staining. **b)** Representative gel-based assays for PAPP-A2 truncation mutants. 1.5-fold, serial dilutions of full length or truncated PAPP-A2 were used as indicated by the green ramp.

a)

```
Q9BXP8|PAPP-A2 1012 QDDRSEQPEGSLKKEDEVWLKVCFNRPGEARAIFIFLTTDGLVPGEHQQPTVTLYLTDVRGSNHSLGTYGLSCQHNPLIINVTHHQNVLF
Q13219|PAPP-A   998 Q-----------LAQTTFWLRAYFSQPMVAAAVIVHLVTDGTYYGDQKQETISVQLLDTKDQSHDLGLHVLSCRNNPLIIPVVHDLSQPF
consensus           *...........      **.  *  *  * *. . * ***   *.  * *... * * .   * **   ***  ***** * *     *
```

PAPP-A M2 Anchor Peptide Binding

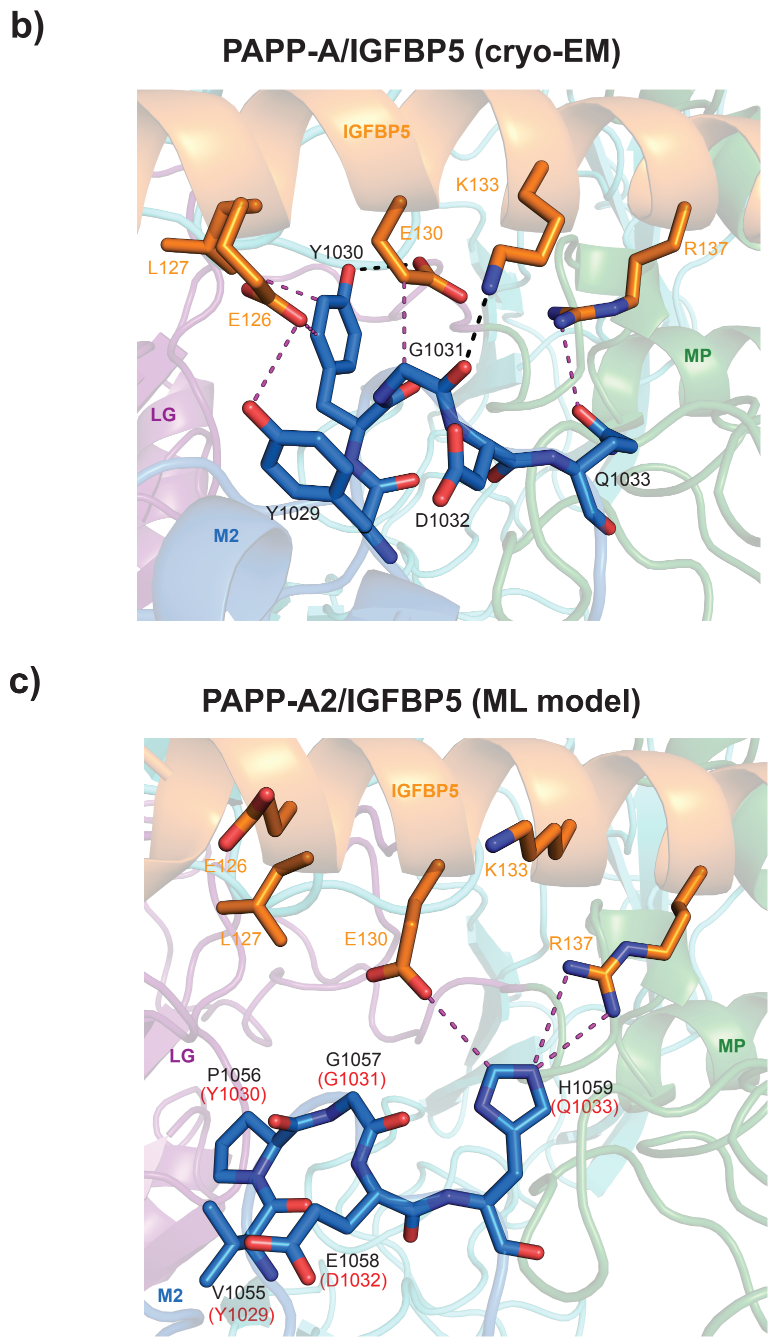

Supplementary Figure 13. Comparison of M2 domain residues involved in IGFBP5 anchor peptide binding in PAPP-A and PAPP-A2

a) Sequence alignment of the M2 domain for PAPP-A2 and PAPP-A with residues involved in anchor peptide binding in PAPP-A boxed in orange. **b)** Zoomed-in view of PAPP-A M2 domain residues involved in anchor peptide binding from the cryo-EM PAPP-A/IGFBP5 anchor peptide complex (PDB 7ufg). Black dashes represent hydrogen bonds while magenta dashes represent distances < 4.0 Å. **c)** Zoomed-in view of PAPP-A2 M2 domain residues around the anchor peptide from the ML-PAPP-A2/IGFBP5 model. Labels in parentheses and red script indicate corresponding PAPP-A residues based on sequence alignment.

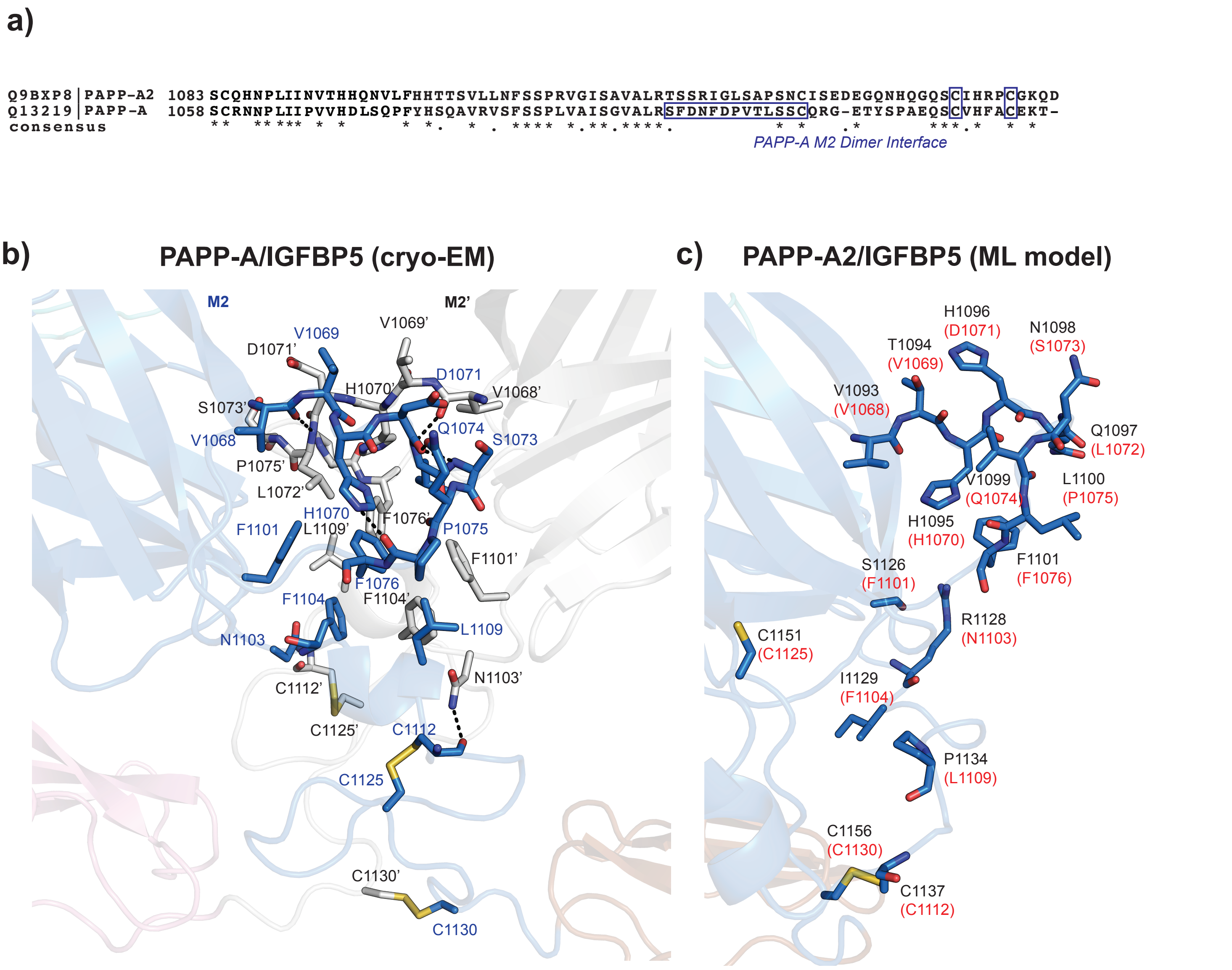

Supplementary Figure 14. Comparison of M2 domain residues involved in dimerization in PAPP-A and PAPP-A2
a) Sequence alignment of the M2 domain for PAPP-A2 and PAPP-A with residues involved in dimerization in PAPP-A boxed in blue. **b)** Zoomed-in view of PAPP-A M2 domain residues responsible for dimerization from the cryo-EM PAPP-A/IGFBP5 anchor peptide structure (PDB 7ufg). One copy of the M2 domain is shown in blue and labeled with normal script while the second copy is shown in white and labeled with prime (') notation. **c)** Zoomed-in view of PAPP-A2 M2 domain residues that correspond to residues responsible for dimerization in PAPP-A from the ML-PAPP-A2/IGFBP5 model. Labels in parentheses and red script indicate corresponding PAPP-A residues based on sequence alignment.

a)

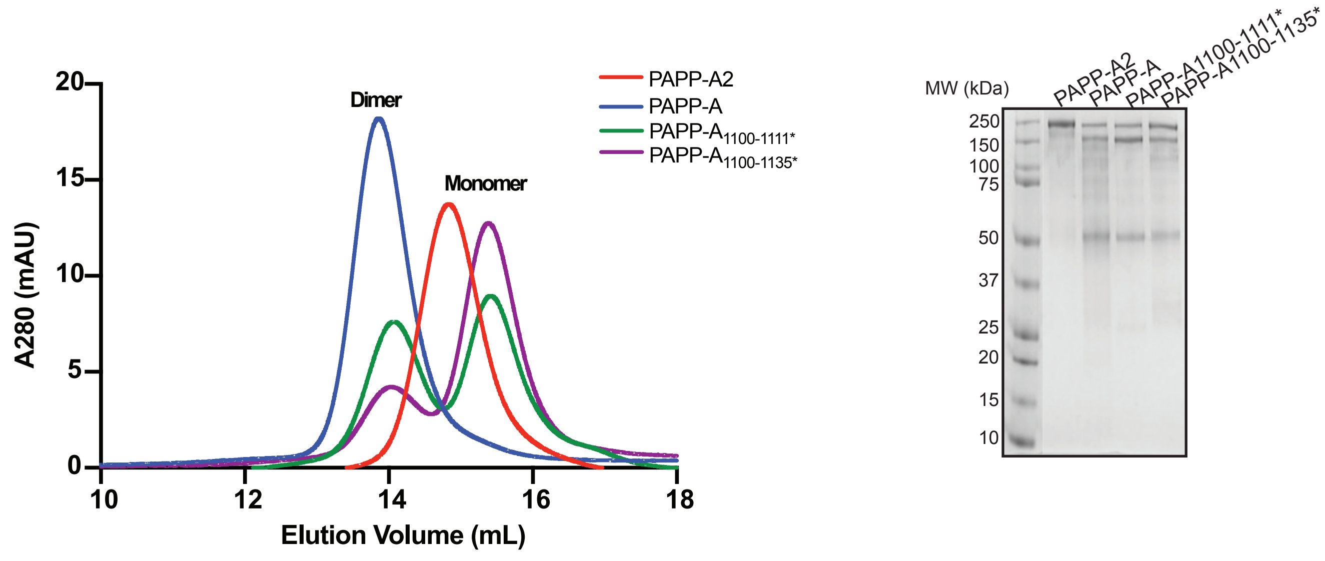

b)

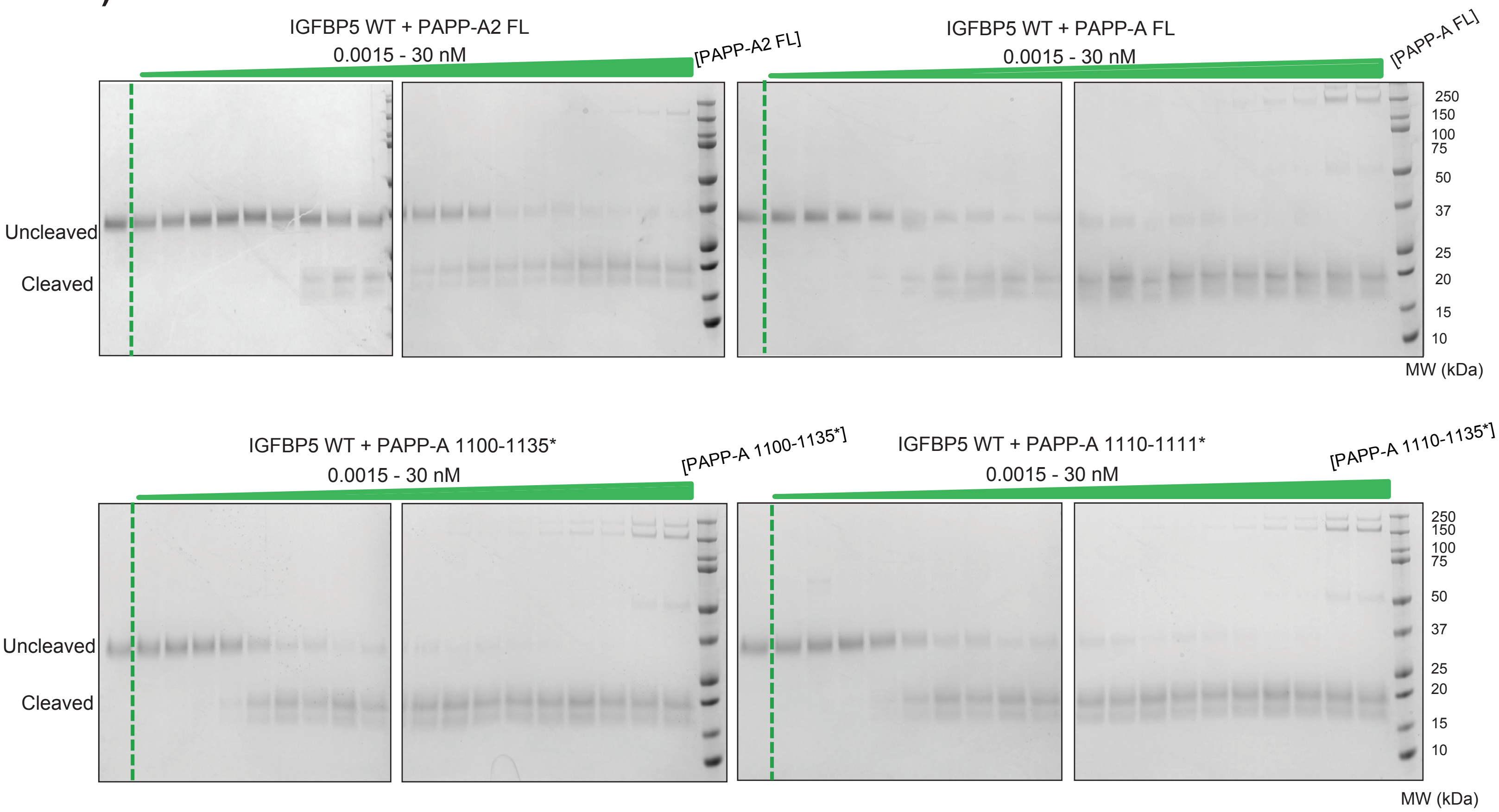

Supplementary Figure 15. Protein quality and representative assay data for PAPP-A/PAPP-A2-M2 domain hybrids

a) Left, final purification step for wild type PAPP-A2 and PAPP-A, and PAPP-A/PAPP-A2-M2 domain hybrid proteins by gel filtration on a Superose 6 Increase 10/300 GL column. Monomeric peak fractions were pooled for PAPP-A2 and PAPP-A/PAPP-A2-M2 domain hybrid proteins for use in assays. The dimeric, wild type PAPP-A peak was pooled for use in assays. Right, protein analysis by SDS-PAGE followed by Coomassie Blue staining. **b)** Representative gel-based assays for PAPP-A2 and PAPP-A, and PAPP-A/PAPP-A2-M2 domain hybrid proteins. 1.5-fold, serial dilutions of PAPP-A2 or PAPP-A were used as indicated by the green ramp.

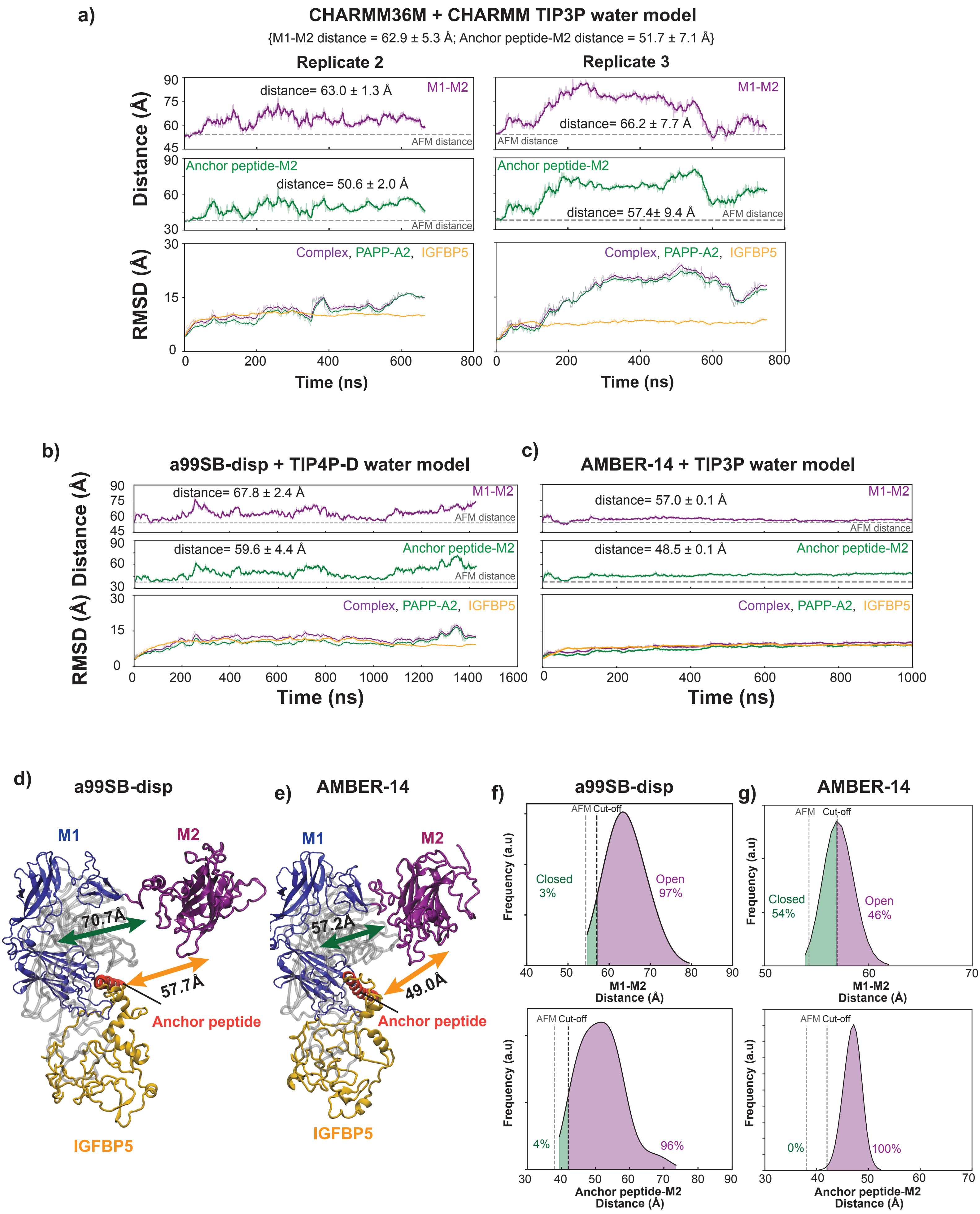
a)
CHARMM36M + CHARMM TIP3P water model
{M1-M2 distance = 62.9 ± 5.3 Å; Anchor peptide-M2 distance = 51.7 ± 7.1 Å}
Replicate 2
Replicate 3
Distance (Å)
RMSD (Å)
distance= 63.0 ± 1.3 Å
M1-M2
AFM distance
distance= 66.2 ± 7.7 Å
Anchor peptide-M2
distance= 50.6 ± 2.0 Å
distance= 57.4± 9.4 Å
Complex, PAPP-A2, IGFBP5
Time (ns)
b)
a99SB-disp + TIP4P-D water model
distance= 67.8 ± 2.4 Å
distance= 59.6 ± 4.4 Å
c)
AMBER-14 + TIP3P water model
distance= 57.0 ± 0.1 Å
distance= 48.5 ± 0.1 Å
d)
a99SB-disp
M1
M2
70.7Å
57.7Å
Anchor peptide
IGFBP5
e)
AMBER-14
57.2Å
49.0Å
f)
a99SB-disp
g)
AMBER-14
AFM
Cut-off
Frequency (a.u)
Closed 3%
Open 97%
Closed 54%
Open 46%
M1-M2 Distance (Å)
4%
96%
0%
100%
Anchor peptide-M2 Distance (Å)

Supplementary Figure 16. Characterizing the structural dynamics of PAPP-A2 in complex with IGFBP5 protein with MD simulations using different force fields

a) CHARMM36M force field with the CHARMM TIP3P water model. **b)** a99SB-disp force field with TIP4P-D water model. **c)** AMBER-14 with TIP3P water model. For each force field, graphs depict MD simulation results for two replicates. Conformational changes in PAPP-A2, crucial for substrate recognition, were evaluated based on the distances between the M1 domain (center of mass of C alpha residues 610-927) and the M2 domain (center of mass of C alpha residues 953-1160), as well as the M2 domain and the anchor peptide (center of mass of C alpha residues 120-143). RMSD values were also measured relative to the AlphaFold multimer (AFM) construct at the start of the simulations (t=0 ns). Exponential moving averages over the trajectory are shown as solid lines. For each replicate, average and standard deviation were determined through block averaging, using the final 400 ns frames of MD simulations with the CHARMM36M force field, 1200 ns frames of MD simulation with the a99SB-disp force field, and 900 ns frames of MD simulations with AMBER-14 to mitigate initial transients. We used blocks of 50 ns frames for measurement. **d)** Open conformation of PAPP-A2 obtained using a99SB-disp force field at t=1400 ns. **e)** Open conformation of PAPP-A2 obtained using AMBER-14 force field at t=1000 ns. **f)** Distribution histogram for a99SB-disp force field. **g)** Distribution histogram for AMBER-14 force fields. For f) and g) the distance between M2 and M1 domains is shown in the top panel and the distance between the M2 domain and the anchor peptide is shown in the bottom panel. Histograms are based on the last 1200 ns, and 900 ns frames of production simulations with a99SB-disp and AMBER-14 force fields, respectively. To estimate distance distributions, a Gaussian kernel density estimator was applied using Python's scikit-learn package, using a covariance factor of 3 Å for a99SB-disp force field and 1 Å for AMBER-14. The AlphaFold multimer (AFM) distance is shown as dashed lines in the plots. The cut-off distances were selected exactly from those calculated for the simulations with the CHARMM36M force field for fair comparison, where the cut-off was determined as the AFM distance + 0.5 * standard deviation.

a)

```
                                             1
IGFBP5     1 -----MVLLTAVLLLLA--------AYAGPAQSLGSFVHCEPCDEKALSMCPPSPLGC-E
IGFBP3     1 MQRARPTLWAAALTLLVLLRGPPVARAGASSAGLGPVVRCEPCDARALAQCAPPPAVCAE
                   .* :*.* **.          .. : .** .*:***** :**: * * *  * *
                                        1

IGFBP5    27 LVKEPGCGCCMTCALAEGQSCGVYTERCAQGLRCLPRQDEEKPLHALLHGRGVCLNEKSY
IGFBP3    34 LVREPGCGCCLTCALSEGQPCGIYTERCGSGLRCQPSPDEARPLQALLDGRGLCVNASAV
             **:*******:****:*** **:*****..**** *  ** :**:***.***:*:* .:

                                                                   K128
IGFBP5    89 REQVKIER-------DSREHEEPT-------TSEMAEETYSPKIFRPKHTRISELKAEAV
IGFBP3    94 SRLRAYLLPAPPAPGNASESEEDRSAGSVESPSVSSTHRVSDPKFHPLHSKIIIIKKGHA
              .             :: * **          *  : .  *   *:* *::*  :*   .

IGFBP5   133 KKDRRKKLTQSKFVGGAENTAHPRIISAPEMRQESEQGPCRRHMEASLQELKASPRMVPR
IGFBP3   154 KDSQRYKVDY-------ESQSTDTQNFSSESKRETEYGPCRREMEDTLNHLKFLNVLSPR
             *..:* *:         *. :      : * ::*:* *****.** :*:.**    : **

IGFBP5   194 AVYLPNCDRKGFYKRKQCKPSRGRKRGICWCVDKYGMKLPGMEYVD-GDFQCHTFDSSNV
IGFBP3   207 GVHIPNCDKKGFYKKKQCRPSKGRKRGFCWCVDKYGQPLPGYTTKGKEDVHCYSMQSK--
             .*::****:*****:***:**:*****:********  ***    .  *.:*::::*.

IGFBP5   254 E
IGFBP3       -
```

b)

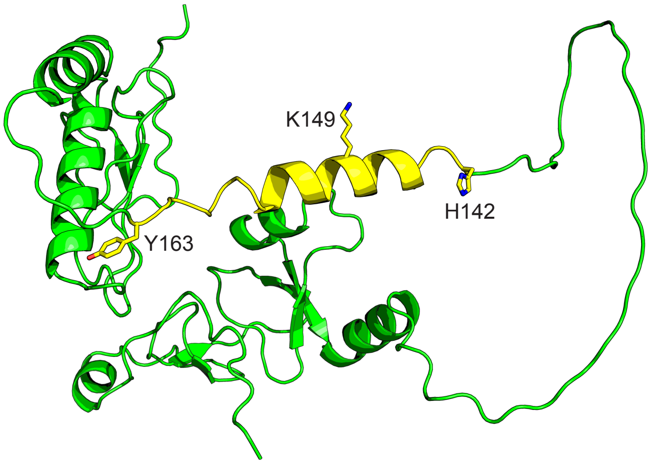

Supplementary Figure 17. Comparison of IGFBP5 and IGFBP3

a) IGFBP5 (Uniprot ID P24593) and IGFBP3 (Uniprot ID P17936) sequences were aligned with Clustal Omega (version 1.2.4). Asterisks under the sequences indicate residues that are conserved, and dots represent residues that are similar. Numbering for the mature protein sequences, which is used in the text, is shown as black script whereas numbering for immature signal peptide and propeptide regions is shown as white script with black highlight. Anchor peptide regions are boxed in green. The location of residue K128 of IGFBP5 is shown in red for reference. **b)** AlphaFold predicted model of IGFBP3 (AF-P17936-F1) with the signal peptide region removed. The putative anchor peptide region is shown in yellow. Note, the putative anchor peptide region has a pLDDT <50 (very low), indicating that prediction of this region should be treated with caution.

Supplementary Table 1. Cryo-EM data collection, refinement, and validation statistics

	PAPP-A2<br>(EMDB-40571)<br>(PDB 8SL1)
**Data collection and processing**	
Magnification	105,000
Voltage (kV)	300
Electron exposure (e–/Å^2)	50
Defocus range (μm)	-1.0 to -2.0
Pixel size (Å)	0.669
Symmetry imposed	C1
Initial particle images (no.)	1,853,196
Final particle images (no.)	186,048
Map resolution (Å)	3.13
FSC threshold	0.143
Map resolution range (Å)	2.4 to 4.0
**Refinement**	
Initial model (AlphaFold code)	AF-Q9BXP8-F1
Model resolution (Å)	3.13
FSC threshold	0.143
Model resolution range (Å)	2.4 to 4.0
Map sharpening *B* factor (Å^2)	-140
Model composition	
Non-hydrogen atoms	6608
Protein residues	843
Ligands	1 Zn, 2 Ca, 3 NAG
B factors (Å^2)	
Protein	57.36
Ligand	42.51
Water	49.69
R.m.s. deviations	
Bond lengths (Å)	0.004
Bond angles (°)	1.008
Validation	
MolProbity score	1.82
Clashscore	8.71
Poor rotamers (%)	0.68
Ramachandran plot	
Favored (%)	94.97
Allowed (%)	5.03
Disallowed (%)	0
