# Supplementary material for: Cryo-EM structure of human PAPP-A2 and mechanism of substrate recognition
Source: Commun Chem. 2023 Oct 28;6:234. doi: 10.1038/s42004-023-01032-y (PMC10613257; doi:10.1038/s42004-023-01032-y)
Supplement: Supplementary file 3 — Description of Additional Supplementary Files [file 42004_2023_1032_MOESM3_ESM.pdf]

Description of Additional Supplementary Files

File name: Supplementary Data 1

Description: PDB validation report

File name: Supplementary Data 2

Description: Sourcing raw data for the all the figures

File name: Supplementary Data 3

Description: PDB file of PAPP-A2 cryo-EM structure
